# Benchmarking the Urology Practice

**DOI:** 10.1155/2008/729204

**Published:** 2008-12-18

**Authors:** Paul A. Brower

**Affiliations:** Orange County Urology Associates Inc., 25200 La Paz Road, Suite 200, Laguna Hills, CA 92653, USA

## Abstract

The medical practice today is relentlessly challenged by medical progress, by rising costs, and by the mounting pressures of the managed care environment. It should be the approach of every medical practice manager and practitioner to seek out and measure up to the best standards so as to optimize patient care and business outcomes. This requires the resolute pursuit of good models, brought about by the fostering of key collaborative relationships that are both practical and strategic. Integral to this process is benchmarking: the way by which information is obtained from both internal and external sources to determine and set the standards for performance. Benchmarking is an invaluable strategic tool.

## 1. INTRODUCTION

The AUA Practice Management Committee sees the broad applicability of benchmarking for the practicing urologist and can conceivably become a clearing house for factual collaboration. Yet, there is no movement in the wider urology community to connect practices toward this effort. To promote involvement, a solution is needed that will help sell the efficacy of benchmarking as a strategic tool for driving growth and improvement. The difficulty, however, is physician buy-in. It is within the nature of the average practitioner to stay focused primarily on patient encounters and to remain preoccupied within the practice. There is also a natural reluctance to share information. Confidentiality and legal issues are usually of great concern and can hamper open dialogue.

While agreements can be drafted to establish parameters and the usefulness of comparing practices is commonly evident, what remains at the heart of the problem is the perception that this is a labor-intensive exercise and an altogether complicated and expensive process.

## 2. MATERIALS AND METHODS

Orange County Urology Associates (OCUA) is a nine-physician, single-specialty group located in South Orange County California that began gathering benchmark data four years ago. OCUA was formed after a merger of two preexisting groups approximately four years ago. We began gathering internal benchmarking data as a means of measuring the success of the merger. We quickly realized the value of the multiple benchmarks that we began to track, and have gradually increased the number of variables that we examine. Our ongoing objective is to understand our capabilities for maximizing profit while dealing with quality care and time management issues. Thus, our approach is not to look at things in a broad or general way but to seek out a careful explanation for each indicator, so as to gain knowledge and provide for actionable results. This requires a process that expands incrementally, one step at a time.

There are two forms of benchmarking: internal and external, with the former always the necessary first step. The process of benchmarking must begin with a baseline exam of the internal data points of the practice: cost and revenue indicators as well as for productivity and staffing. This is the beginning point of reference against which things can be measured and compared and brought to focus. OCUA tracks patient encounter data. We look at every physician, comparing each to their own historical data and to one another. We also benchmark group performance and track it on a semiannual basis for a comparison to previous years. The collection and organization of encounter data give us knowledge about the competing demands for quality patient care, resources, and accountability. Patient mix, patterns of coding, and a myriad of encounter complexities specifically related to physician performance are measured in addition to capitation reporting and claims submission.

## 3. RESULTS

Data for new and return patients is analyzed on a per-physician basis to monitor current procedural terminology (CPT) coding practices in order to determine the accuracy in matching services to the correct code. By creating a matrix with the number of encounters by CPT code for both new and return visits, it becomes immediately apparent if one physician's coding pattern deviates from the others in the group. During this review, we have found both overcoding and undercoding through the random selection of charts for individual physicians. These particular charts are then reviewed by a collective group and analysis is undertaken comparing the chart documentation to the billing slip. In the vast majority of cases, the consensus is that the coding and billing were accurate. Sometimes, a small addition to the documentation would have allowed a higher service code, and on a few occasions, it was felt that the level of service was not justified. Inaccurate documentation can mean heavy legal and financial consequences for the practice, so it has been important to know how and why individual physicians are using inaccurate codes. We find that most of these errors occur because of either a misunderstanding of the guidelines, bad habits, or as a result of the difficulty in correlating CPT codes with clinical service. We have used this review as an audit tool and learning experience.

The inoffice procedure is the economic kicker for the practice. It drives income. We look at all procedures done in the office, noting the ratio of procedures per new and return patient. Once again, the comparisons are made on an individual and group basis, and we find that physicians with subspecialty interest, such as incontinence, will generally have higher numbers for procedures, and thus, higher collections.

Hospital activities are tracked separately. To insure that everyone carries an equal burden, each physician is reviewed for the work done in hospital consultations and followup visits. The number of inhospital surgical procedures is tracked per physician, with regard to new and return patient work, including the number of long cases by CPT that take the physician out of the office for a half day. Hospital work is valuable to the group practice, but, in comparison to office work, compensation for assisting in the hospital is so poor per unit of time spent that we pay assisting physicians two thirds of the amount collected by the primary surgeon. To wrestle with this problem, we set aside a pool of money to compensate the primary surgeon and to promote collegial cooperation for work that would not, otherwise, be equitable to the individual.

Billing charges are also tracked on an individual and group basis, but though they hold some comparative value for what physicians do, the charges are variable from year to year and by payer class. They are reviewed more in relation to what really matters, which is dollars, collected. Another way to measure physician activity is through the use of relative value units (RVUs), because, based on time and activity formulas, they are not only a more objective measure for physician productivity, but, being that the RVU amount is assigned to each service via CPT codes, they also have a direct relationship with billing compliance. Because reimbursement per CPT code varies greatly by the geographic location of the practice, RVUs may be the only way to compare productivity between practices and individuals in different regions.

At the end of the day, what really matters economically is collections. We track these on an individual and group basis, monthly and semiannually, and notice that the ratio of collections to billed charges tends to vary little among individuals, unlike the individual payer mix, which can reveal marked differences in the ratio of collections to billed charges and indicate the need for contract renegotiation or even outright termination.

Comparisons between office-generated and hospital-generated collections are startling. It is well known that reimbursement for surgical procedures has plummeted. Yotan calculates office fees increased 51% while surgical fees decreased 28% from 1995 to 2004 [1]. As a group, OCUA generates only 15% of its income from the operating room, and this percentage is remarkably reproducible over four years, varying only from 14.5% to 16%; individuals track from 7% to 23%, with the highest level representing income generated by our male infertility specialist who is handsomely rewarded in cash for surgical procedures. By using these data, we are able to objectively determine the cruciality of the office work relative to the financial health of the practice. Understanding its significance in comparison to hospital time enables us to deduce how to best spend our time, without sacrificing quality care. We now see the average income per patient encounter as well as per surgical procedure and can look at income per unit of time, comparing the office to the hospital. We are using a half day as the basic scheduling unit, and, consequently, it is exceptional that anyone leaves the office to do a case in the middle of a block. To that end, individuals scheduled for the hospital round on all the patients for the practice before or between cases to preserve the scheduling unit time commitment; those that are in the office stay in the office. It was only when we had analyzed this objective and reproducible data for our income that we really came to understand how we make our money. This is a powerful example of how benchmarking has modified physician behavior and enhanced productivity.

While planning the space and designing of our new office, we looked at two years of encounter data for each physician for everyday. We analyzed the number of patients each MD saw per half day and how many office procedures they did per session. We counted the procedures that required a larger room such as a prostate biopsy or cystoscopy which allowed us to most accurately predict the number of exam and procedure rooms that were required. The data showed that 1 procedure room and 2 exam rooms per physician were sufficient for our patient volume. This is an eye-opener for those individuals previously overestimating space requirements. The office is configured for 23 exam/procedure rooms, with room now for 2 urodynamic setups, 3 ultrasounds, and capacity for numerous endoscopic and bladder scanning procedures. Encounter data for the first two years in the new facility shows that increases in exams, procedures, and income are substantial. The 23 rooms allow 7 MDs and a PA to work simultaneously.

## 4. CONCLUSION

In conclusion, we find that quantifiable information which is based on data analysis rather than subjectivity can allow for physician behavior modification and unnecessary predilections. The physicians in the OCUA responded quite favorably to this information when it was presented to them in a transparent manner. The numbers do not lie. Time spent developing a panel of internal benchmarks and tracking them regularly enables us to identify clear patterns, integrate critical functions, reduce inefficiencies, and improve the fitness of our practice.

Given this success, we are hopeful to see an increase in outreach activities among urology practices so that we can begin the process of external benchmarking. Originality is great, but there is limitless value in learning from others.

## References

[1] Lotan Y, Cadeddu JA, Roehrborn CG, Stage KH (2004). The value of your time: evaluation of effects of changes in medicare reimbursement rates on the practice of urology. *The Journal of Urology*.

